# Air pollution dispersion from biomass stoves to neighboring homes in Mirpur, Dhaka, Bangladesh

**DOI:** 10.1186/s12889-019-6751-z

**Published:** 2019-04-23

**Authors:** Anne M. Weaver, Emily S. Gurley, Christina Crabtree-Ide, Henrik Salje, Eun-Hye Yoo, Lina Mu, Nasrin Akter, Pavani K. Ram

**Affiliations:** 1 Department of Epidemiology and Environmental Health, University at Buffalo, Buffalo, NY USA; 2 Programme for Emerging Infections, icddr,b, Dhaka, Bangladesh; 3 Department of Epidemiology, Johns Hopkins Bloomberg School of Public Health, Baltimore, MD USA; 4 Mathematical Modelling of Infectious Diseases Unit, Institut Pasteur, Paris, France; 5 Department of Geography, University at Buffalo, Buffalo, NY USA

**Keywords:** Air pollution, Biomass stove, Fine particulate matter, Carbon monoxide, Bangladesh

## Abstract

**Background:**

Indoor air pollution, including fine particulate matter (PM_2.5_) and carbon monoxide (CO), is a major risk factor for pneumonia and other respiratory diseases. Biomass-burning cookstoves are major contributors to PM_2.5_ and CO concentrations. However, high concentrations of PM_2.5_ (> 1000 μg/m^3^) have been observed in homes in Dhaka, Bangladesh that do not burn biomass. We described dispersion of PM_2.5_ and CO from biomass burning into nearby homes in a low-income urban area of Dhaka, Bangladesh.

**Methods:**

We recruited 10 clusters of homes, each with one biomass-burning (index) home, and 3–4 neighboring homes that used cleaner fuels with no other major sources of PM_2.5_ or CO. We administered a questionnaire and recorded physical features of all homes. Over 24 h, we recorded PM_2.5_ and CO concentrations inside each home, near each stove, and outside one neighbor home per cluster. During 8 of these 24 h, we conducted observations for pollutant-generating activities such as cooking. For each monitor, we calculated geometric mean PM_2.5_ concentrations at 5-6 am (baseline), during biomass burning times, during non-cooking times, and over 24 h. We used linear regressions to describe associations between monitor location and PM_2.5_ and CO concentrations.

**Results:**

We recruited a total of 44 homes across the 10 clusters. Geometric mean PM_2.5_ and CO concentrations for all monitors were lowest at baseline and highest during biomass burning. During biomass burning, linear regression showed a decreasing trend of geometric mean PM_2.5_ and CO concentrations from the biomass stove (326.3 μg/m^3^, 12.3 ppm), to index home (322.7 μg/m^3^, 11.2 ppm), neighbor homes sharing a wall with the index home (278.4 μg/m^3^, 3.6 ppm), outdoors (154.2 μg/m^3^, 0.7 ppm), then neighbor homes that do not share a wall with the index home (83.1 μg/m^3^,0.2 ppm) (*p* = 0.03 for PM_2.5_, *p* = 0.006 for CO).

**Conclusion:**

Biomass burning in one home can be a source of indoor air pollution for several homes. The impact of biomass burning on PM_2.5_ or CO is greatest in homes that share a wall with the biomass-burning home. Eliminating biomass burning in one home may improve air quality for several households in a community.

**Electronic supplementary material:**

The online version of this article (10.1186/s12889-019-6751-z) contains supplementary material, which is available to authorized users.

## Background

The World Health Organization (WHO) estimates that 4.3 million people die every year from exposure to indoor air pollution, with 1.7 million of these deaths occurring in the South East Asian region alone [1]. Residents of Dhaka, Bangladesh experience high levels of indoor and ambient air pollution exposure [2–4], as well as a high burden of disease from acute lower respiratory infection (ALRI) [5].

Fine particulate matter (PM_2.5_), suspended particles in the air of 2.5 μm or less in diameter, and carbon monoxide (CO) are common indicators of indoor air pollution in low-income settings because they are relatively easy to measure and have known health effects [6, 7]. PM_2.5_ has been implicated in contributing to ALRI [8, 9] and other respiratory illnesses [10], including in Bangladesh [11]. The negative health impact of CO exposure includes low birth weight, delayed behavioral development of infants and children, exacerbation of chronic cardiovascular and pulmonary conditions, and, in extreme cases, coma or death [12, 13]. Household sources of indoor PM_2.5_ and CO include combustion of biomass fuel (wood, bamboo, charcoal, agricultural residue), which can be exacerbated by the use of poor quality biomass-burning stoves for cooking or heating, and cigarette smoking [6, 14–16]. In addition, ventilation, ambient air pollution, and building materials may affect indoor air quality in a home [3, 17–19].

Currently, interventions to reduce indoor air pollution exposure from cooking focus on individual homes. However, if biomass fuel burning in one home affects the air quality in neighboring homes, community-wide interventions may be necessary to sufficiently improve indoor air quality. In Mirpur, a low-income area of Dhaka, Bangladesh, PM_2.5_ concentrations were observed to exceed 1000 μg/m^3^ for a mean of 35 min per day in homes that exclusively use cleaner fuels (natural gas, electricity) for cooking [4]. These high concentrations of PM_2.5_ are consistent with concentrations produced from burning biomass fuels [4, 20]. Peaks in PM_2.5_ concentrations in homes that exclusively used cleaner cooking fuels occurred at similar times to those in biomass-burning homes, indicating that indoor PM_2.5_ may result from neighborhood cookstove sources [4]. Similarly, Chowdhury et al. [7] have observed high ambient PM_2.5_ and CO concentrations in an urban area in Bangladesh, particularly during cooking times. Although biomass burning is well recognized as a source of indoor air pollution in homes, the effects of biomass burning on neighboring homes are not well understood.

In this observational cohort study, we aimed to describe the dispersion of PM_2.5_ and CO from biomass cookstove sources into neighboring homes and determine the contribution of neighbors’ biomass burning stoves to PM_2.5_ and CO concentrations within homes, in a low-income urban area in Mirpur, Dhaka, Bangladesh.

## Methods

### Study population

**Fig. 1:**
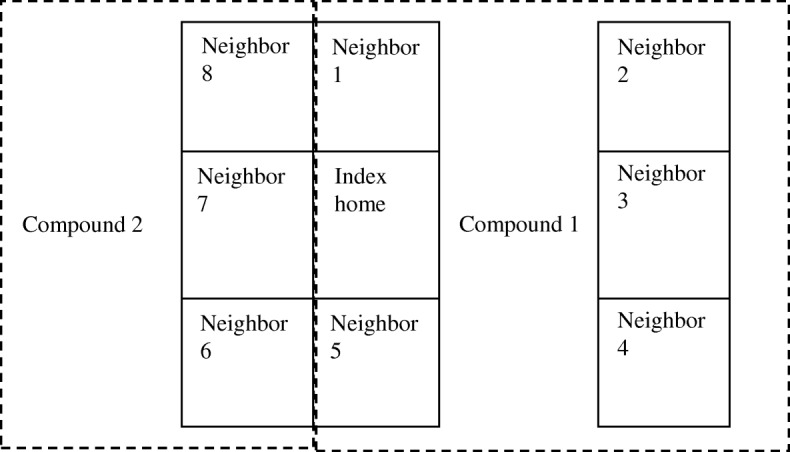
Typical layout of compounds in Mirpur, with biomass-burning index homes and neighbor homes

This investigation was conducted in a densely populated (35,200 people/km^2^, 2011) low-income area of Mirpur, Dhaka, Bangladesh, where the type of cooking fuel used is heterogeneous [3]. Mirpur homes are typically arranged in compounds, with several one-room homes immediately adjacent to each other, a shared latrine, and a small central courtyard or walkway; compounds are often adjacent to other compounds with shared walls (Fig. 1).

### Eligibility criteria

We recruited clusters of index and neighbor homes. Index homes exclusively used biomass fuels (dung, crop residue/grass, rice husk, dried leaves, coal, charcoal, wood, and/or bamboo) and a traditional stove for cooking. Only one index home was recruited per compound. Once the index home was identified, we recruited three to four neighbor homes, defined as homes immediately surrounding the index home in any direction that primarily used one of the following cleaner fuels for cooking: natural gas, biogas, electricity, and/or kerosene. Index and neighbor homes located on an upper level (above ground level) of an apartment building were ineligible, because of potential variation in sources, levels, and movement of air pollutants on upper levels of a multi-story building compared to ground level. Eligible respondents intended to reside in their current home for the subsequent one week. If more than four neighbor homes were eligible, we prioritized recruiting those neighboring homes that had main entrances nearest to the main entrance of the index home. It is possible that other nearby homes that we did not recruit may have used biomass-burning stoves.

Once eligible index and neighbor homes were identified, we sought informed consent from the adult who usually cooks in each household. As many participants were unable to read, a consent form was read aloud to them, and they were asked to either sign or provide a thumbprint as a mark of consent. The first ten clusters of households (index and neighbor homes) visited that met all eligibility criteria and consented to participate were enrolled. The study protocol was approved by the institutional review boards at icddr,b and the University at Buffalo. No interventions were performed, therefore this is not registered as a trial.

### Data collection

After obtaining written informed consent, study staff administered a questionnaire to each participant to elicit information on demographics, stove type and location, and behaviors that may affect exposure to air pollution, including cigarette smoking, ventilation behavior (opening and closing windows and doors, turning fans on and off), cooking fuel use, and burning substances other than for cooking. The participant was then asked to show his/her home and cooking space. Study staff measured the size of the main sleeping space of the home using a measuring tape. Air quality in the main sleeping space serves as a proxy for individual exposure, as many homes consist of only one room: the sleeping space where family members spend substantial time. Staff recorded the location of the main cooking area, including distance from the home to the cooking area (if external to the home) and the distance from index to neighbor homes in steps (approximately 0.4 m per step). Study staff drew sketches of each cluster, including the index home and all neighboring homes of the index home, including participants and non-participants. Locations of primary stoves, walls, doors, and windows were indicated on the sketch.

After administering the baseline questionnaire and taking measurements of dimensions of the home, study staff installed two sets of PM_2.5_ and CO monitors for each index and neighbor home: one as close as possible to the primary stove, and one inside the main sleeping space, above the participant’s bed. Additional monitors were placed at one outdoor location per cluster. The outdoor monitors were located between the main entrances of the index home and neighbor home(s), under a roof or eave and out of plain sight, in order to protect instruments from rain and theft. Due to the variety in structure of local homes, we were unable to standardize the locations of these outdoor monitors. PM_2.5_ and CO monitors recorded measurements once per minute for at least 24 h. We used University of California, Berkeley Particle Monitors (University of California, Berkeley, Berkeley, CA, USA) to measure PM_2.5_ concentrations and Lascar CO monitors (Lascar Electronics, Salisbury, UK) to measure CO concentrations. The lower limit of detection of the University of California, Berkeley Particle Monitors is 50 μg/m^3^, which is twice the WHO-recommended 24-h mean PM_2.5_ concentration of ≤25 μg/m^3^ [2, 3, 10, 21–23]. The lower limit of detection for Lascar CO monitors is 0 ppm, with a resolution of 0.5 ppm. The WHO recommended 24-h mean CO concentration is ≤6.11 ppm [24].

Study staff conducted detailed observations of household activities for approximately 8 h on the working day following the initial interview, concurrent with PM_2.5_ and CO monitoring. Study staff recorded cooking activities and fuels used, ventilation activities, smoking, and burning substances other than for cooking (e.g. for fragrance, lighting, mosquito control, or garbage disposal). Study staff was stationed near each index home in order to prioritize recording activities occurring at the index home. However, they moved about the cluster as needed in order to record activities occurring at the participating neighbor homes. Residents were asked to continue normal daily activities as much as possible while monitoring was taking place.

### Analysis

We calculated geometric mean PM_2.5_ and CO concentrations at each monitor location for the following times: between 5 and 6 am (baseline), during observed biomass cooking in the index home, the observation period during which no biomass cooking occurred in any home, and 24 h of monitoring time. Previous research has shown that the lowest PM_2.5_ concentrations are observed in Mirpur from 5 to 6 am [4], so the 5–6 am hour served as a baseline for this study. We used data from observations to identify biomass cooking times in the index home. Although neighbor homes primarily used cleaner fuels, our observations indicated that some biomass fuel use occurred when cleaner fuels were unavailable. For our measure of biomass cooking time in an index home, we excluded any times during which an enrolled neighbor home was also observed to cook with biomass fuel.

We used ANOVA to describe whether geometric mean PM_2.5_ and CO concentrations varied by monitor location at the pre-specified times. Our independent variable was location of the monitor, categorized as follows: near the index stove, in the index home’s main sleeping space, monitors (near the stove and in the main sleeping space) of neighbor homes that share a wall with the index home, outdoor monitors, and monitors (near the stove and in the main sleeping space) of neighbor homes that do not share a wall with the index home. Although PM_2.5_ and CO are expected to dissipate differently, we still expected that the order of dissipation would be the same, that is, highest concentrations for both pollutants would be highest in cooking space of biomass homes, then living space, then neighbor homes with a shared wall, then outside, then neighbor homes with no shared wall. Therefore, the ranking of environments is the same for both pollutants. We added a constant of 0.001 for all CO values in order to calculate the geometric mean, which requires nonzero values; the lowest possible value of the geometric mean CO concentration was therefore 0.001 ppm. We substituted a value equal to half the limit of detection for all PM_2.5_ readings at or below the limit of detection, a common method of analyzing data from instruments with high limits of detection [25]. Therefore, the lowest possible value of the geometric mean PM_2.5_ concentration was 25 μg/m^3^. We have provided a supplemental table comparing results using this method to results substituting a value equal to the limit of detection for all PM_2.5_ readings at or below the limit of detection (Additional file 5).

Using the PM_2.5_ and CO measurements at the index stove monitor as the referent category, we used linear regression to describe the relationship between the location of a monitor (index home, neighbor home—shared wall, outdoor, neighbor home—no shared wall) and geometric mean PM_2.5_ and CO concentrations during index biomass cooking and 24 h of monitoring. We examined the following as potential confounders: building material(s) of the home, having a smoker living in the home, distance to the index home (in steps), area of the home, having a secondary biomass stove, ambient temperature, and relative humidity. Covariates that changed the bivariate measure of association by 10% were retained in the final models. We examined ventilation of the home (presence of at least one window in the home) and location of the index stove (indoor or outdoor) as potential effect modifiers by stratifying results by these factors.

We also performed spatial variogram analysis to describe the relationship between geometric mean PM_2.5_ and CO concentrations in index and neighbor homes, limited to index stove cooking times, and Euclidian distance from a PM_2.5_ or CO source (the biomass-burning stove). Variograms are used to describe variability in PM_2.5_ or CO concentrations as a function of the distance between the source of pollution (biomass stove) and the monitor.

## Results

### Demographics

We recruited a total of 44 homes for this study (10 index homes and 34 neighboring homes). Index homes and neighboring homes shared many similar characteristics, including smoking in the home, low education levels, and high proportion of homes with only one room (Table 1). Neighbor homes were more likely to have a window compared to index homes. Most cooking areas were located inside the main housing structure, but some were located in a separate room from the sleeping space. Separation of the sleeping space from the stove was more common in index compared to neighbor homes; four of ten index home stoves were located outside. The most common type of biomass fuel used in index homes was wood (*n* = 9), followed by small sticks of wood or bamboo (*n* = 1). Supplemental fuels used in index stoves included: paper (*n* = 6), kerosene (*n* = 5), wood (n = 5), plastic (*n* = 3), and small sticks of wood or bamboo (*n* = 2). Although neighbor homes almost exclusively used electricity as their primary cooking fuel, 21 of 34 had a secondary biomass stove, likely used when electricity was unavailable [3].

**Table 1:** Descriptive characteristics for index and neighbor homes (*N* = 44)

	Index Homes (*n* = 10)	Neighbor Homes (*n* = 34)
	N (%)	N (%)
Female respondent	10 (100)	32 (94)
Any smoking in the home	3 (30)	11 (32)
Less than 1 year education	7 (70)	23 (68)
Location of primary stove		
Inside main living space	2 (20)	26 (76)
Attached room	4 (40)	6 (18)
Unattached room	0 (0)	2 (6)
Outside	4 (40)	0 (0)
Type of primary stove		
Biomass	10 (100)	0 (0)
Kerosene	0 (0)	2 (6)
Electricity	0 (0)	31 (94)
Respondent uses secondary stove	3 (30)	27 (79)
Type of secondary stove		
Biomass	0 (0)	24 (80)
Kerosene	2 (67)	2 (7)
Electricity	1 (33)	1 (3)
Respondent uses secondary stove more than once per week	1 (33)	13 (48)
Home has only one room	8 (80)	29 (85)
Building material of home		
Thatch	2 (20)	3 (9)
Brick/Concrete	0 (0)	21 (62)
Multiple materials	8 (80)	10 (29)
At least one window in home	3 (30)	20 (59)
	Mean (SD)	Mean (SD)
Number of people in home	6 (2.2)	5.5 (2.4)
Age of respondent (years)	38.8 (10.7)	35.5 (10.1)
Area of home in m^2^, mean (SD)	9.8 (3.7)	10.0 (2.8)
Monthly household income (USD)	135.2 (48.3)	160.1 (155.6)

### Cooking and ventilation events

We observed cooking events, window opening events, door opening events, and fan use events over approximately eight hours of structured observation for each cluster (Table 2). Cooking patterns varied between index and neighbor homes. Of 84 observed cooking events, 17 (20%) were biomass cooking events, 13 (76%) of which occurred in nine of ten index homes (we did not observe cooking in one index home). We observed 65 electricity cooking events (77% of cooking events), all of which occurred in neighbor homes*.* The median duration of cooking events with electricity (65 min) was considerably shorter than for those using biomass fuel (137 min), however, the total amount of time spent cooking was similar among the two fuel groups (median of 189 and 185 min for electric and biomass stoves). Homes that cooked with electricity tended to cook several times during the observation period, whereas most of those using biomass fuel only cooked once. We observed no smoking or burning of substances other than for cooking during structured observations in either index or neighbor homes.

**Table 2:** Descriptive characteristics of events observed during structured observation (median 500 min observation)

Type of event	Total number of events observed	Number of events per household^a^	Duration per event (minutes)^a^	Total duration of events of this type per household (minutes)^a^
		Median (IQR)	Median (IQR)	Median (IQR)
Index homes (*n* = 10)				
Cooking				
Biomass	13	1 (1)	135 (87)	185 (48)
Kerosene	1	1 (NA)	14 (NA)	14 (NA)
Window opening	3	1 (0)	508 (110)	508 (110)
Door opening	12	1 (0)	482 (158)	490 (20)
Turning fan on	32	3 (1)	111 (110)	381 (100)
Neighbor homes (*n* = 34)				
Cooking				
Biomass	4	1 (0)	50 (55)	50 (109)
Electricity	65	2 (2)	68 (93)	189 (123)
Kerosene	1	1 (NA)	105 (NA)	105 (NA)
Window opening	20	1 (1)	495 (20)	495 (20)
Door opening	59	1 (1)	295 (380)	495 (34)
Turning fan on	97	3 (1)	115 (127)	404 (104)

^a^Descriptive statistics of events reported only for homes in which that event was observed

Participants from index and neighbor homes had similar ventilation behaviors. Twenty-three participants had a single window. Of these, twenty-two were kept open during the entire structured observation with the remaining window kept open for 292 min (nearly five hours). Likewise, doors were open for the vast majority (482 min, approximately six hours) of the structured observation period. Fan use events were the most frequently observed events (median 111 min among index homes, 115 min among neighbor homes); many participants turned their fans on and off throughout the day. Participants had their fans on for a median of over six hours during the eight-hours observation periods in both index and neighbor homes. All participants who had a window kept it open during cooking, and almost all participants had their doors open (*n* = 42, 95%) and fans on (*n* = 38, 86%) during cooking.

### PM_2.5_ and CO measurements

We monitored PM_2.5_ and CO for a total of 95 locations, at least 24 h each, from 44 households. Due to equipment error, five PM_2.5_ recordings had missing data for 12 or more hours. Additionally, one index stove produced extremely high concentrations of PM_2.5_ and CO (over 10 times the mean of other homes during biomass cooking), so we removed the monitors from that home from our analyses. Data analysis was performed using the data from the remaining 88 PM_2.5_ recordings and 93 CO recordings. In one cluster, we did not observe biomass cooking in the index home during our observation period. For this cluster, we estimated index cooking time based on elevated CO concentrations in the index home at a common cooking time during the day prior to observation, as biomass cooking was the only obvious source of sustained elevated CO concentrations for all homes in the study.

At baseline (5-6 am), geometric mean concentrations at all monitor locations were at the lowest possible values for both PM_2.5_ (25 μg/m^3^) and CO (0.001 ppm) (Table 3). During index stove biomass cooking events, increased geometric mean PM_2.5_ concentrations were recorded at all monitors in all homes. Geometric mean concentrations of PM_2.5_ during index stove biomass cooking events were highest at the index stove (mean 326.3, SD 211.6 μg/m^3^), followed by the sleeping space in the index home (mean 322.7, SD 408.1 μg/m^3^), neighbor home that shares a wall with the index home (mean 278.4, SD 423.7 μg/m^3^), outdoor (mean 154.2, SD 80.6 μg/m^3^), then neighbor home that does not share a wall with the index home (mean 83.1, SD 49.2 μg/m^3^) (ANOVA *p*-value = 0.005)—Additional file 3 shows PM_2.5_ concentrations for one cluster during biomass cooking. Concentrations of CO during index stove biomass cooking events were also highest at the index stove (mean 12.3, SD 13.2 ppm), followed by index home (mean 11.2, SD 18.8 ppm), neighbor home that shares a wall with the index home (mean 3.6, SD 7.3 ppm), outdoor (mean 0.7, SD 1.3 ppm), then neighbor home that does not share a wall with the index home (mean 0.2, SD 0.6 ppm) (ANOVA *p*-value< 0.0001)—Additional file 4 shows CO concentrations for one cluster during biomass cooking. During observation periods with no biomass cooking, we observed no differences in PM_2.5_ concentrations at different locations (*p* = 0.6), although CO concentrations were slightly lower at outdoor locations and in neighbor homes that did not share a wall with the index home (*p* = 0.1). During 24 h of monitoring, average geometric mean PM_2.5_ and CO concentrations were also highest at the index stove, then in the index home and outdoor, then in neighbor homes (p = 0.1 for PM_2.5_, *p* = 0.007 for CO). There were no differences in 24-h PM_2.5_ and CO concentrations between neighbor homes with and without a shared wall. Additional file 5 compares PM_2.5_ concentrations 1) substituting readings at or below the limit of detection with half of the limit of detection (25 μg/m^3^). as in the main analysis with 2) substituting readings at or below with limit of detection with the limit of detection (50 μg/m^3^). Baseline and 24-h means were about 25 μg/m^3^ higher than in the main analysis, as expected, but the difference between the two methods was not as great during index stove cooking and non-cooking times. Associations of PM_2.5_ concentration by stove location were similar using both methods.

**Table 3:** Geometric mean PM_2.5_ and CO concentrations at selected times and locations^a^

Location of monitor	At baseline (5-6 am)	During index stove cooking	Non-cooking time	24 h
	Mean (SD)	Mean (SD)	Mean (SD)	Mean (SD)
Geometric mean PM_2.5_ (μg/m^3^)^a^ (*N* = 88 monitors)				
Index stove (*n* = 8)^b^	29.8 (8.3)	326.3 (211.6)	62.9 (32.7)	61.5 (27.3)
Index home (*n* = 9)^b^	28.4 (28.4)	322.7 (408.1)	54.2 (18.7)	51.9 (16.8)
Neighbor home—shared wall (*n* = 18)	27.6 (5.1)	278.4 (423.7)	51.4 (15.4)	47.1 (10.0)
Outdoor (*n* = 8)	33.2 (10.9)	154.2 (80.6)	70.7 (25.9)	57.1 (19.9)
Neighbor home—no shared wall (*n* = 44)	30.0 (7.4)	83.1 (49.2)	63.5 (40.5)	47.5 (14.9)
*p*-value (ANOVA)	0.5	0.005	0.6	0.1
Geometric mean CO (ppm)^a^ (*N* = 93 monitors)				
Index stove (*n* = 9)^b^	0.001 (0)	12.3 (13.2)	0.02 (0.04)	0.02 (0.03)
Index home (*n* = 9)^b^	0.002 (0.002)	11.2 (18.8)	0.03 (0.08)	0.03 (0.04)
Neighbor home—shared wall (*n* = 18)	0.001 (0.00003)	3.6 (7.3)	0.02 (0.05)	0.01 (0.05)
Outdoor (*n* = 9)	0.001 (0)	0.7 (1.3)	0.003 (0.003)	0.006 (0.008)
Neighbor home—no shared wall (*n* = 48)	0.001 (0.00002)^b^	0.2 (0.6)	0.002 (0.002)	0.006 (0.01)
*p*-value (ANOVA)	0.8	< 0.0001	0.1	0.007

^a^Lowest possible values are 25 μg/m^3^ for PM_2.5_ concentration and 0.001 ppm for CO concentration

^b^One high outlier excluded

Multivariable linear regression models to examine the association between location of the monitor and PM_2.5_ and CO concentrations included two covariates: distance to the index home in steps (index homes had a value of 0 steps) and the presence of a secondary biomass stove in the home. We did not account for smoking in the home as no smoking was observed to take place during biomass cooking times. In exploratory analyses, PM_2.5_ and CO concentrations were not substantially different during electricity cooking periods compared to periods of no cooking, and kerosene cooking events were few, so we did not account for electricity or kerosene cooking in our models.

**Fig. 2:**
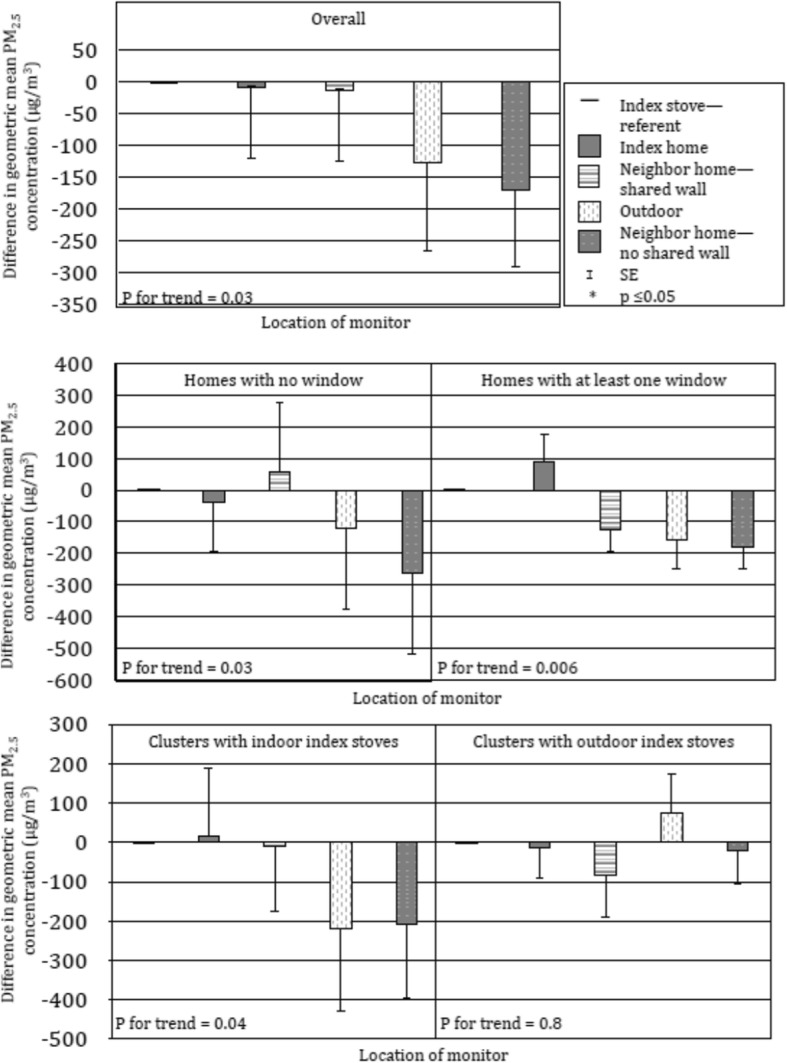
Association between monitor location and PM_2.5_ concentrations during biomass cooking. (N = 88)^1^. ^1^ Findings from linear regression, adjusted for distance to index home (in steps) and presence of a secondary biomass stove

**Fig. 3:**
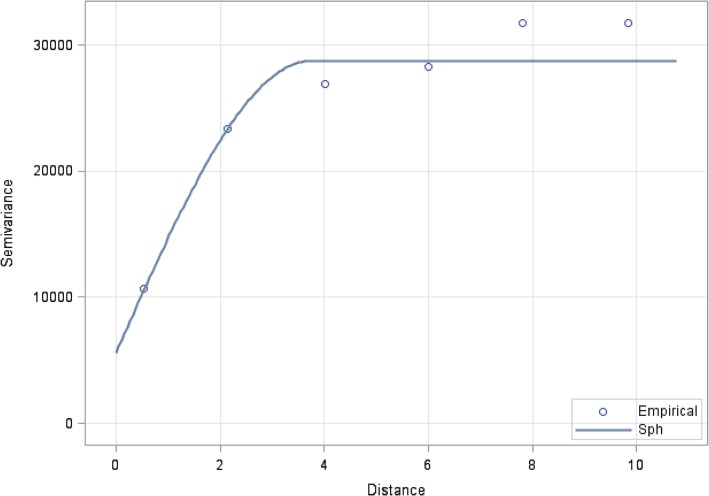
Spherical model variogram of PM_2.5_ concentrations over distance from biomass stove (m) during biomass cooking

During index stove biomass cooking events, we observed a trend of lower geometric mean PM_2.5_concentrations with monitor locations further from the index stove overall (p for trend 0.03) (Fig. 2 and Additional file 1). In stratified models, we did observe a trend of lower geometric mean PM_2.5_ in clusters with no windows in the index home (p for trend 0.03), but this trend was clearer in clusters with at least one window in the index home (p for trend = 0.006), particularly for neighbor homes that share a wall with the index stove. We observed a trend similar to the overall data in clusters with indoor index stoves (p for trend 0.04), but not in clusters with outdoor index stoves (p for trend 0.8). We did not observe any associations between location of the monitor and 24-h geometric mean PM_2.5_ concentrations (Additional file 2). Consistent with spatial dependence in PM_2.5_ from biomass burning, variogram analysis of PM_2.5_ concentrations showed a steadily increasing semivariance in PM_2.5_ up to distances of around five meters from the biomass stove, suggesting that spatial dependence in PM_2.5_ was limited at further distances (Fig. 3).

**Fig. 4:**
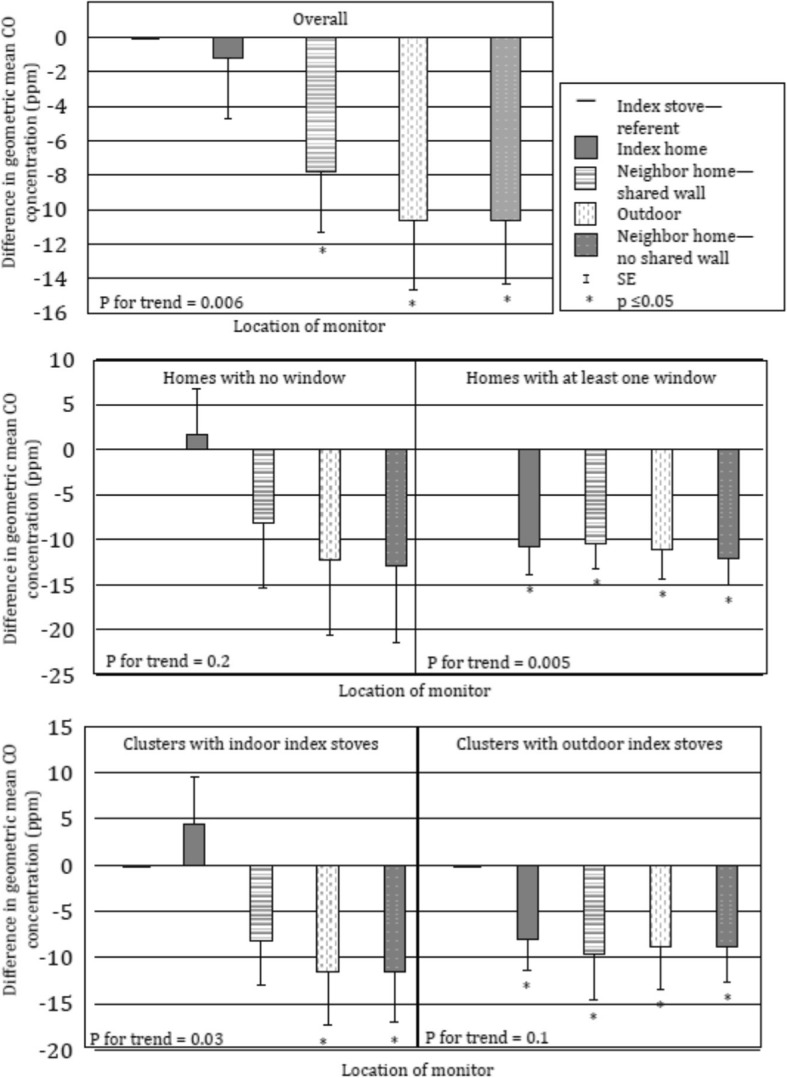
Association between monitor location and CO concentrations during biomass cooking (N = 88)^1^. ^1^ Findings from linear regression, adjusted for distance to index home (in steps) and presence of a secondary biomass stove

**Fig. 5:**
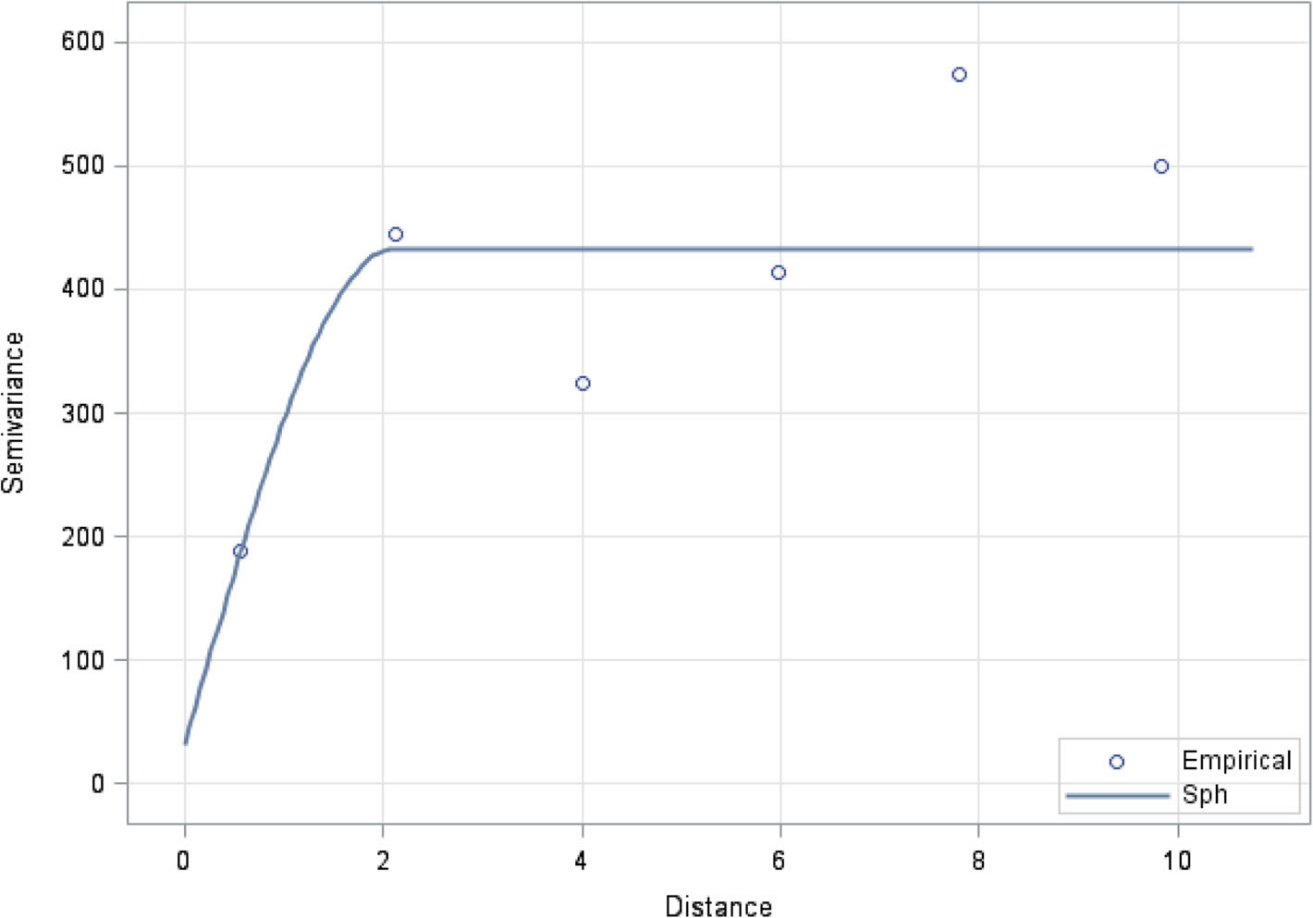
Spherical model variogram of CO concentrations over distance from biomass stove (m) during biomass cooking

Reduced CO concentrations during index stove cooking were associated with location of monitor relative to index stove (p for trend 0.006) overall, in homes with at least one window (p for trend 0.005), and in clusters with indoor index stoves (p for trend 0.03) (Fig. 4 and Additional file 1). Geometric mean CO concentrations were very low, 0.03 ppm or less, over 24 h. However, we did observe a trend of lower CO concentrations at monitoring locations further from the index stove overall (p for trend 0.1) and in homes with at least one window (p for trend 0.1), though not significant (Additional file 2). Semivariance appeared to increase at distances beyond than that observed with PM_2.5_, consistent with the lighter CO particles travelling farther than PM_2.5_ (Fig. 5).

## Discussion

In this study of dispersion of air pollution from biomass stoves, we observed elevated concentrations of PM_2.5_ and CO in homes that did not use biomass fuel and had no other obvious source of air pollution, other than being near households that burned biomass for cooking. PM_2.5_ and CO concentrations were highest near biomass cookstoves while biomass fuel was burned, thirteen times the WHO guideline for PM_2.5_ and ten times the WHO recommendations for CO. Among neighboring homes, the effect of biomass cooking on PM_2.5_ and CO concentrations was greater in homes that shared a wall with an index biomass-burning home compared to outdoor concentrations and concentrations in neighbor homes that did not share a wall with the index home. Compared to non-cooking times, during biomass cooking, neighbor homes with a shared wall had more than a five-fold increase in PM_2.5_ concentrations and over 100-fold increase in CO concentrations. Neighbor homes with no shared walls had about a 31% increase in PM_2.5_ and about 100-fold increase in mean CO concentrations. Variogram analyses indicated PM_2.5_ and CO may travel several meters, far enough away from a biomass stove to affect a neighboring home, as home areas averaged 10 m^2^.

Similar to our findings, previous studies have demonstrated better air quality in individual homes using cleaner fuels compared to those using biomass fuels [3, 4, 18, 26, 27]. Our results also indicate that if a home switches from biomass to cleaner fuels but neighbors continue to use biomass, the residents of the cleaner fuel-burning home may still be at risk for exposure to high air pollution levels during cooking times. Mean air pollutant concentrations in homes that used clean fuel but shared a wall with biomass-burning homes were eleven and eight times the WHO standards for PM_2.5_ and CO, respectively. These results are most applicable to densely populated urban areas, where residents live in close proximity and biomass fuel use is common. Therefore, we recommend future cleaner fuel initiatives to target entire communities, rather than individual homes. Residents of homes that use cleaner fuels are often wealthier than those that use biomass fuels [18, 19, 28], so future clean fuel initiatives may need to consider equity-promoting strategies for poor households to switch to cleaner fuels. The primary cleaner fuel source used in this community was electricity. However, our prior research in Dhaka has demonstrated inconsistencies in electricity supply, with frequent power cuts lasting several hours to several days [29]. In this study, we observed a high proportion of neighboring homes using secondary biomass stoves, possibly for use when electricity is not available. Although there are air quality benefits to those who primarily, but not always, use cleaner fuels, a consistent supply of cleaner fuels would likely provide maximum benefit.

Our results suggest that windows may modify the effect of biomass burning on homes’ PM_2.5_ and CO concentrations; neighboring homes with a window had a stronger trend of decreasing PM_2.5_ and CO as their locations were further removed from the index stove compared to those with no window. Previous studies in Honduras [30] and Bangladesh [18, 31] have demonstrated inverse associations between PM concentrations and ventilation in a home, suggesting a PM clearance role of ventilation. We also observed participants opening windows and doors for much of the day during the warm study months of August and September. This would be most useful to clearing pollutants from a home that uses biomass fuels. Only about half of the participants in this study had at least one window in their home. Our prior work in Dhaka suggests that installation of one window may be a feasible method to improve air quality within a home, but perceptions of theft risk and lack of physical space for a window may be barriers [29].

The location of the biomass stove may modify the dispersion of PM_2.5_ and CO to neighboring homes; we saw clearer associations between location of monitors and the location of index stove in compounds with an indoor index stove compared to those with an outdoor index stove. PM_2.5_ and CO concentrations decreased where the index stove was inside compared to those where the stove was outside. Biomass-burning homes could benefit from having an outdoor stove, but this could adversely affect air quality in neighboring homes. Even if the stove was indoors, air quality in the sleeping space was better than it was near the stove, possibly due to ventilation in the home or the location of the stove relative to the sleeping area.

A limitation of this study was the inability to account for sources of ambient and indoor air pollution other than biomass cooking within the cluster. In Mirpur, indoor PM_2.5_ concentrations appear to be influenced by ambient sources of air pollution [3], including industrial sources and motor vehicle traffic. Nearby industries are expected to affect the air quality in neighboring homes fairly equally. The study area was not near any major roads, and local roads were typically travelled primarily on foot or tricycle rickshaw, so motor vehicle traffic was unlikely to be a major source of ambient air pollution. Therefore, biomass fuel was likely to be the primary source of variance in indoor air pollution measured in index and neighboring homes. Another limitation of this study was the high limit of detection, 50 μg/m^3^, of the University of California, Berkeley air quality monitors. Our estimates of PM_2.5_ concentrations may have been erroneously high, particularly during periods of relatively low PM_2.5_. However, we were able to examine very high (≥200 μg/m^3^) concentrations of PM_2.5_ associated with biomass cooking. In addition, relative humidity may affect the accuracy of PM_2.5_ measurements, but adjustment for relative humidity did not substantially change our estimates and the UCB monitors did not show systematic bias in laboratory tests [21, 23]. Using an alternate method, substituting the limit of detection for readings at or below the limit of detection, did not result in substantially different associations. However, the mean PM_2.5_ concentrations, especially at baseline and over 24 h, may be inaccurate.

Additionally, this study was conducted in August and September, the end of the hot and rainy season, when PM_2.5_ concentrations are lowest in Bangladesh [3]. This study may not accurately reflect air pollution dynamics throughout the year. This study was conducted in a densely populated area, and results may not be applicable to less densely populated areas.

## Conclusions

In this study, we found that biomass cooking can adversely affect air quality in homes adjacent to biomass-burning homes. As expected, we observed that PM_2.5_ and CO concentrations were highest near biomass stoves and in homes that use biomass as a primary cooking fuel. However, we also observed increased concentrations in air pollutants in neighboring homes, particularly homes that share a wall with biomass-burning homes. Interventions to improve access to cleaner fuels should focus on entire communities, not just individual homes. Doing so may necessitate addressing within-community barriers to adoption and sustained utilization of cleaner fuels.

## Additional files

Additional file 1: **Table S1.** Associations between monitor location and PM_2.5_ and CO concentrations during biomass cooking (*N* = 88)^1^. (DOCX 19 kb)

Additional file 2: **Table S2.** Associations between monitor location and PM_2.5_ and CO concentrations during 24 h of monitoring (N = 88)^1^. (DOCX 19 kb)

Additional file 3: **Figure S1.** Effects of index stove biomass cooking on PM_2.5_ concentrations (μg/m^3^) at various locations in a representative cluster. (DOCX 76 kb)

Additional file 4: **Figure S2.** Effects of index stove biomass cooking on carbon monoxide concentrations (ppm) at various locations in a representative cluster.^1^. ^1^ Carbon monoxide concentrations did not rise above 0 ppm in a neighbor home that does not share a wall with the index home during this time. (DOCX 61 kb)

Additional file 5: **Table S3.** Geometric mean PM_2.5_ substituting limit of detection concentrations at selected times and locations (N = 88). (DOCX 14 kb)

## Abbreviations

ALRI: Acute lower respiratory infection
ANOVA: ANalysis Of VAriance
CO: Carbon monoxide
PM_2.5_: Fine particulate matter, 2.5 μm in diameter or smaller
SD: Standard deviation
WHO: World Health Organization

## References

[1] World Health Organization (2014). Burden of disease from Household Air Pollution for 2012: Summary of results.

[2] Gurley ES, Homaira N, Salje H, Ram PK, Haque R, Petri W, Bresee J, Moss WJ, Breysse P, Luby SP, Azziz-Baumgartner E (2013). Indoor exposure to particulate matter and the incidence of acute lower respiratory infections among children: a birth cohort study in urban Bangladesh. Indoor Air.

[3] Gurley ES, Salje H, Homaira N, Ram PK, Haque R, Petri WA Jr, Bresee J, Moss WJ, Luby SP, Breysse P, et al. Seasonal concentrations and determinants of indoor particulate matter in a low-income community in Dhaka, Bangladesh. Environ Res. 2013;121:11–6. 10.1016/j.envres.2012.10.004 PMC3582809 23127494

[4] Salje H, Gurley ES, Homaira N, Ram PK, Haque R, Petri W, Moss WJ, Luby SP, Breysse P, Azziz-Baumgartner E. Impact of neighborhood biomass cooking patterns on episodic high indoor particulate matter concentrations in clean fuel homes in Dhaka, Bangladesh. Indoor Air. 2014;24(2):213–20. 10.1111/ina.12065 PMC3932152 24033488

[5] Gurley ES, Salje H, Homaira N, Ram PK, Haque R, Petri WA, Bresee J, Moss WJ, Luby SP, Beysse P, Azziz-Baumgartner E (2014). Indoor exposure to particulate matter and age at first acute lower respiratory infection in a low-income Urban Community in Bangladesh. Am J Epidemiol.

[6] Smith KR, McCracken JP, Thompson L, Edwards R, Shields KN, Canuz E, Bruce N. Personal child and mother carbon monoxide exposures and kitchen levels: methods and results from a randomized trial of woodfired chimney cookstoves in Guatemala (RESPIRE). J Expo Sci Environ Epidemiol. 20(5):406–16. 10.1038/jes.2009.30 PMC4575221 19536077

[7] Chowdhury Z, Le LT, Al Masud A, Chang KC, Alauddin M, Hossain M, Zakaria ABM, Hopke PK (2012). Quantification of indoor air pollution from using Cookstoves and estimation of its health effects on adult women in Northwest Bangladesh. Aerosol Air Qual Res.

[8] Holgate ST, Samet JM, Koren HS, Maynard RL (1999). Air pollution and health.

[9] McGrath JJ, Barnes CD (1982). Air pollution--physiological effects.

[10] World Health Organization (2006). WHO air quality guidelines for particulate matter, ozone, nitrogen dioxide and sulfur dioxide.

[11] Ram PK: An observational study of indoor air pollution on pneumonia and influenza in an urban slum in Bangladesh In: American Society of Tropical Medicine and Hygiene Annual Meeting. Atlanta, GA; 2010.

[12] Carbon Monoxide (1999). Environmental Health Criteria.

[13] Dix-Cooper L, Eskenazi B, Romero C, Balmes J, Smith KR (2012). Neurodevelopmental performance among school age children in rural Guatemala is associated with prenatal and postnatal exposure to carbon monoxide, a marker for exposure to woodsmoke. NeuroToxicology.

[14] Ezzati M, Saleh H, Kammen DM (2000). The contributions of emissions and spatial microenvironments to exposure to indoor air pollution from biomass combustion in Kenya. Environ Health Perspect.

[15] Clark ML, Peel JL, Burch JB, Nelson TL, Robinson MM, Conway S, Bachand AM, Reynolds SJ (2009). Impact of improved cookstoves on indoor air pollution and adverse health effects among Honduran women. Int J Environ Health Res.

[16] Halder AK, Gurley ES, Naheed A, Saha SK, Brooks WA, El Arifeen S, Sazzad HMS, Kenah E, Luby SP (2009). Causes of early childhood deaths in urban Dhaka, Bangladesh. PLoS ONE [Electronic Resource].

[17] Dasgupta S, Huq M, Khaliquzzaman M, Pandey K, Wheeler D (2006). Who suffers from indoor air pollution? Evidence from Bangladesh. Health Policy Plann.

[18] Dasgupta S, Huq M, Khaliquzzaman M, Pandey K, Wheeler D (2006). Indoor air quality for poor families: new evidence from Bangladesh. Indoor Air.

[19] Dasgupta S, Wheeler D, Huq M, Khaliquzzaman M (2009). Improving indoor air quality for poor families: a controlled experiment in Bangladesh. Indoor Air.

[20] Siddiqui AR, Lee K, Bennett D, Yang X, Brown KH, Bhutta ZA, Gold EB. Indoor carbon monoxide and PM2.5 concentrations by cooking fuels in Pakistan. Indoor Air. 19(1):75–82. 10.1111/j.1600-0668.2008.00563.x 19076247

[21] Edwards R, Smith KR, Kirby B, Allen T, Litton CD, Hering S (2006). An inexpensive dual-chamber particle monitor: laboratory characterization. J Air Waste Manage Assoc.

[22] Berkeley Air Monitoring Group IAPT, School of Public Health, University of California, Berkeley, USA: UCB Particle Monitor User Manual. In*.*; 2008.

[23] Chowdhury Z, Edwards RD, Johnson M, Shields KN, Allen T, Canuz E, Smith KR (2007). An inexpensive light-scattering particle monitor: field validation. J Environ Monit.

[24] World Health Organization Regional Office for Europe: WHO guidelines for indoor air quality: selected pollutants. 2010. 23741784

[25] Hewett P, Ganser GH (2007). A comparison of several methods for analyzing censored data. Ann Occup Hyg.

[26] Bautista LE, Correa A, Baumgartner J, Breysse P, Matanoski GM (2009). Indoor charcoal smoke and acute respiratory infections in young children in the Dominican Republic. Am J Epidemiol.

[27] Khalequzzaman M, Kamijima M, Sakai K, Chowdhury NA, Hamajima N, Nakajima T (2007). Indoor air pollution and its impact on children under five years old in Bangladesh. Indoor Air.

[28] Emmelin A, Wall S (2007). Indoor air pollution: a poverty-related cause of mortality among the children of the world. Chest.

[29] Weaver AM, Parveen S, Goswami D, Crabtree-Ide C, Rudra C, Yu J, Mu L, Fry AM, Sharmin I, Luby SP (2017). Pilot intervention study of household ventilation and fine particulate matter concentrations in a low-income urban area, Dhaka, Bangladesh. Am J Trop Med Hyg.

[30] Clark ML, Reynolds SJ, Burch JB, Conway S, Bachand AM, Peel JL (2010). Indoor air pollution, cookstove quality, and housing characteristics in two Honduran communities. Environ Res.

[31] Crabtree-Ide CR, Carole B. Rudra, Benjamin J. Silk, Dhiman Dutt, Saumil S. Doshi, Jaynal Abedin, Doli Goswami, W. Abdullah Brooks, Alicia Fry, Stephen P. Luby, Adam L. Cohen, Pavani K. Ram: Household factors associated with indoor air pollution in a low-income urban area in Bangladesh. In: American Society of Tropical Medicine and Hygiene Annual Meeting: December 5, 2011 2011; Philadelphia, PA; 2011.

