# Supplementary material for: Air pollution dispersion from biomass stoves to neighboring homes in Mirpur, Dhaka, Bangladesh
Source: BMC Public Health. 2019 Apr 23;19:425. doi: 10.1186/s12889-019-6751-z (PMC6480710; doi:10.1186/s12889-019-6751-z)
Supplement: Supplementary file 1 — Table S1. Associations between monitor location and PM2.5 and CO concentrations during biomass cooking (N = 88)1. (DOCX 19 kb) [file 12889_2019_6751_MOESM1_ESM.docx]

**Table S1.** Associations between monitor location and PM_2.5_ and CO concentrations during biomass cooking (N=88)^1^

|  | **Overall** | **Homes with no window** | **Homes with at least one window** | **Clusters with indoor index stove** | **Clusters with outdoor index stove** |
| --- | --- | --- | --- | --- | --- |
| **Geometric mean PM_2.5_ (µg/m^3^)^1^** | **β (95% CI)** | **β (95% CI)** | **β (95% CI)** | **β (95% CI)** | **β (95% CI)** |
| Index stove (n=8)^2^ | REF | REF | REF | REF | REF |
| Index home (n=9)^2^ | -17.1  (-236.2, 202.0) | -68.8  (-392.1, 254.6) | 131.0  (-20.5, 282.6) | -11.1  (-350.4, 328.3) | -16.9  (-161.7, 127.9) |
| Neighbor home—shared wall (n=18) | -38.7  (-249.2, 171.8) | -77.2  (-468.6, 312.2) | -74.6  (-188.6, 38.4) | -77.4  (-375.3, 220.5) | -72.6  (-279.3, 134.1) |
| Outdoor (n=8) | -163.7  (-426.0, 98.6) | -263.3  (-740.2, 213.6) | -111.8  (-272.8, 49.3) | -274.4  (-668.6, 119.8) | 42.5  (-146.4, 231.3) |
| Neighbor home—no shared wall (n=44) | -198.0  (-424.3, 28.2) | -397.0  (-868.9, 74.9) | -132.7  (-247.5, -18.0)* | -281.0  (-668.6, 119.8) | -24.4  (-187.8, 139.0) |
| p for trend | 0.03 | 0.03 | 0.006 | 0.04 | 0.82 |
| **Geometric mean CO (ppm)^1^** | **β (95% CI)** | **β (95% CI)** | **β (95% CI)** | **β (95% CI)** | **β (95% CI)** |
| Index stove (n=9)^2^ | REF | REF | REF | REF | REF |
| Index home (n=9)^2^ | -1.2  (-8.0, 5.7) | 1.6  (-8.4, 11.6) | -10.8  (-17.5, -4.0)* | 4.4  (-5.5, 14.3) | -8.1  (-14.7, -1.6)* |
| Neighbor home—shared wall (n=18) | -7.8  (-14.6, -1.0) | -8.1  (-22.2, 6.0) | -10.4  (-15.9, -4.9)* | -8.2  (-17.7, 1.3) | -9.6  (-19.3, 0.03) |
| Outdoor (n=9) | -10.6  (-18.7, -2.6)* | -12.2  (-28.6, 4.2) | -11.1  (-17.6, -4.7)* | -11.6  (-22.8, -0.3)* | -8.9  (-17.7, -0.09)* |
| Neighbor home—no shared wall (n=48) | -10.6  (-17.9, -3.4)* | -12.9  (-29.6, 3.7) | -12.1  (-17.7, -6.5)* | -11.6  (-22.1, -1.0)* | -8.8  (-16.3, -1.3)* |
| p-value (ANOVA) | 0.006 | 0.2 | 0.005 | 0.03 | 0.1 |

^1^Findings from linear regression, adjusted for distance to index home (in steps) and presence of a secondary biomass stove.
