# Supplementary material for: Air pollution dispersion from biomass stoves to neighboring homes in Mirpur, Dhaka, Bangladesh
Source: BMC Public Health. 2019 Apr 23;19:425. doi: 10.1186/s12889-019-6751-z (PMC6480710; doi:10.1186/s12889-019-6751-z)
Supplement: Supplementary file 2 — Table S2. Associations between monitor location and PM2.5 and CO concentrations during 24 h of monitoring (N = 88)1. (DOCX 19 kb) [file 12889_2019_6751_MOESM2_ESM.docx]

**Table S2.** Associations between monitor location and PM_2.5_ and CO concentrations during 24 hours of monitoring (N=88)^1^

|  | **Overall** | **Homes with no window** | **Homes with at least one window** | **Clusters with indoor index stove** | **Clusters with outdoor index stove** |
| --- | --- | --- | --- | --- | --- |
| **Geometric mean PM_2.5_ (µg/m^3^)^1^** | **β (95% CI)** | **β (95% CI)** | **β (95% CI)** | **β (95% CI)** | **β (95% CI)** |
| Index stove (n=8)^2^ | REF | REF | REF | REF | REF |
| Index home (n=9)^2^ | -10.8  (-25.1, 3.6) | -17.4  (-36.2, 1.3) | 3.5  (-18.1, 25.0) | -2.6  (-18.3, 13.0) | -23.3  (-47.6, 1.0) |
| Neighbor home—shared wall (n=18) | -11.7  (-25.5, 2.1) | -16.3  (-39.0, 6.5) | -3.5  (-19.7, 12.7) | -7.5  (-21.2, 6.2) | -9.0  (-43.7, 25.7) |
| Outdoor (n=8) | -0.8  (-17.9, 16.3) | 0.1  (-27.6, 27.8) | 3.4  (-19.4, 26.2) | -6.0  (-24.1, 12.1) | 13.2  (-18.5, 44.9) |
| Neighbor home—no shared wall (n=44) | -7.4  (-22.0, 7.2) | -10.8  (-38.2, 16.6) | -0.7  (-16.8, 15.4) | -8.4  (-23.7, 6.8) | 1.1  (-26.3, 28.5) |
| p for trend | 0.8 | 0.7 | 0.9 | 0.4 | 0.6 |
| **Geometric mean CO (ppm)^1^** | **β (95% CI)** | **β (95% CI)** | **β (95% CI)** | **β (95% CI)** | **β (95% CI)** |
| Index stove (n=9)^2^ | REF | REF | REF | REF | REF |
| Index home (n=9)^2^ | 0.005  (-0.01, 0.02) | -0.006  (-0.02, 0.01) | 0.04  (0.004, 0.08)* | 0.007  (-0.01, 0.03) | 0.001  (-0.03, 0.03) |
| Neighbor home—shared wall (n=18) | -0.006  (-0.02, 0.01) | -0.01  (-0.04, 0.02) | -0.002  (-0.03, 0.03) | 0.007  (-0.01, 0.03) | -0.03  (-0.08, 0.01) |
| Outdoor (n=9) | -0.01  (-0.04, 0.007) | -0.02  (-0.05, 0.01) | -0.009  (-0.04, 0.03) | -0.005  (-0.03, 0.02) | -0.03  (-0.07, 0.01) |
| Neighbor home—no shared wall (n=48) | -0.01  (-0.03, 0.006) | -0.02  (-0.05, 0.01) | -0.009  (-0.04, 0.02) | -0.004  (-0.03, 0.02) | -0.02  (-0.06, 0.01) |
| p-value (ANOVA) | 0.1 | 0.2 | 0.1 | 0.2 | 0.4 |

^1^Findings from linear regression, adjusted for distance to index home (in steps) and presence of a secondary biomass stove.
