# Supplementary figures and images for: Air pollution dispersion from biomass stoves to neighboring homes in Mirpur, Dhaka, Bangladesh
Source: BMC Public Health. 2019 Apr 23;19:425. doi: 10.1186/s12889-019-6751-z (PMC6480710; doi:10.1186/s12889-019-6751-z)

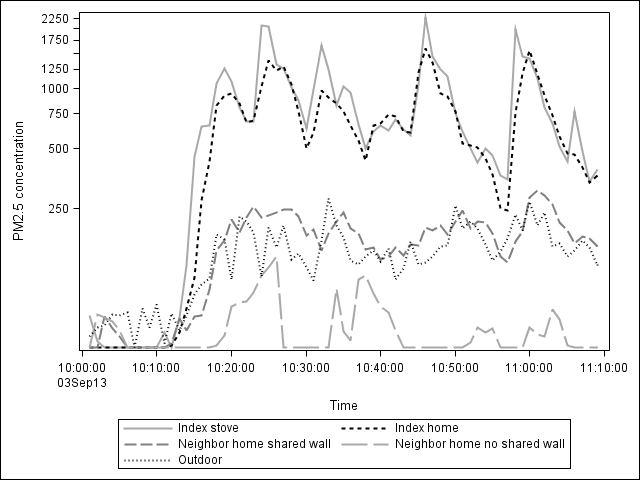


Biomass burning begins in index home

Supplement: Supplementary file 3 — Figure S1. Effects of index stove biomass cooking on PM2.5 concentrations (μg/m3) at various locations in a representative cluster. (DOCX 76 kb) [file 12889_2019_6751_MOESM3_ESM.docx]

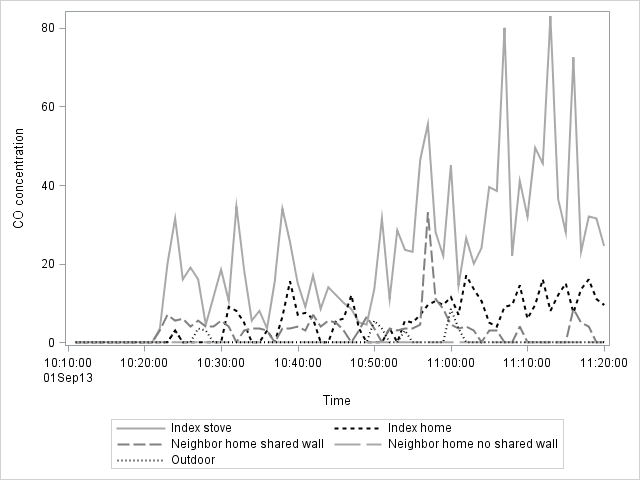


Biomass burning begins in index home

Supplement: Supplementary file 4 — Figure S2. Effects of index stove biomass cooking on carbon monoxide concentrations (ppm) at various locations in a representative cluster.1. 1 Carbon monoxide concentrations did not rise above 0 ppm in a neighbor home that does not share a wall with the index home during this time. (DOCX 61 kb) [file 12889_2019_6751_MOESM4_ESM.docx]
