# Supplementary material for: Air pollution dispersion from biomass stoves to neighboring homes in Mirpur, Dhaka, Bangladesh
Source: BMC Public Health. 2019 Apr 23;19:425. doi: 10.1186/s12889-019-6751-z (PMC6480710; doi:10.1186/s12889-019-6751-z)
Supplement: Supplementary file 5 — Table S3. Geometric mean PM2.5 substituting limit of detection concentrations at selected times and locations (N = 88). (DOCX 14 kb) [file 12889_2019_6751_MOESM5_ESM.docx]

**Table S3.** Geometric mean PM_2.5_ substituting limit of detection concentrations at selected times and locations (N = 88)

| **Location of monitor** | **At baseline**  **(5-6am)** | **During index stove cooking** | **Non-cooking time** | **24 hours** |
| --- | --- | --- | --- | --- |
| **Geometric mean PM_2.5_ (µg/m^3^) using half limit of detection ^1^** | **Mean**  **(SD)** | **Mean**  **(SD)** | **Mean**  **(SD)** | **Mean**  **(SD)** |
| Index stove (n=8) | 29.8  (8.3) | 326.3  (211.6) | 62.9  (32.7) | 61.5  (27.3) |
| Index home (n=9) | 28.4  (28.4) | 322.7  (408.1) | 54.2  (18.7) | 51.9  (16.8) |
| Neighbor home—shared wall (n=18) | 27.6  (5.1) | 278.4  (423.7) | 51.4  (15.4) | 47.1  (10.0) |
| Outdoor (n=8) | 33.2  (10.9) | 154.2  (80.6) | 70.7  (25.9) | 57.1  (19.9) |
| Neighbor home—no shared wall (n=44) | 30.0  (7.4) | 83.1  (49.2) | 63.5  (40.5) | 47.5  (14.9) |
| p-value (ANOVA) | 0.5 | 0.005 | 0.6 | 0.1 |
| **Geometric mean PM_2.5_ (µg/m^3^) using limit of detection^2^** | **Mean**  **(SD)** | **Mean**  **(SD)** | **Mean**  **(SD)** | **Mean**  **(SD)** |
| Index stove (n=8) | 52.2  (3.7) | 339.8  (215.5) | 79.6  (30.9) | 85.4  (26.7) |
| Index home (n=9) | 51.6  (2.5) | 332.1  (403.1) | 74.7  (19.2) | 76.5  (20.0) |
| Neighbor home—shared wall (n=18) | 51.5  (3.5) | 290.7  (430.2) | 70.6  (68.8) | 69.6  (12.8) |
| Outdoor (n=8) | 53.9  (5.0) | 180.3  (105.3) | 89.3  (23.4) | 78.4  (19.3) |
| Neighbor home—no shared wall (n=44) | 52.7  (4.9) | 100.4  (48.6) | 82.9  (41.3) | 70.2  (15.4) |
| p-value (ANOVA) | 0.7 | 0.007 | 0.6 | 0.1 |

^1^Values of PM_2.5_ concentration at or below the limit of detection are imputed as half the limit of detection, 25 µg/m^3^.

^2^Values of PM_2.5_ concentration at or below the limit of detection are imputed as the limit of detection, 50 µg/m^3^.
